# Acute lymphoblastic leukemia with *Fusarium solani* infection: a case report

**DOI:** 10.3389/fmed.2025.1561843

**Published:** 2025-08-01

**Authors:** Bo Liang, Tu Ni, Wenbin Ni, Xu Zhang, Qinqin Ai

**Affiliations:** ^1^Department of Hematology, Hangzhou Red Cross Hospital, Hangzhou, Zhejiang, China; ^2^Department of Ultrasonography, Hangzhou Red Cross Hospital, Hangzhou, Zhejiang, China; ^3^Department of Radiology, Hangzhou Red Cross Hospital, Hangzhou, Zhejiang, China; ^4^Department of Hepatology, Hangzhou Xixi Hospital, Hangzhou, Zhejiang, China

**Keywords:** acute lymphoblastic leukemia, *Fusarium solani*, cutaneous infection, wound debridement, immunocompromise

## Abstract

**Background:**

*Fusarium* infections are rare but life-threatening in immunocompromised patients, particularly those with acute lymphoblastic leukemia (ALL) undergoing chemotherapy. This case report describes a patient with ALL who developed multifocal cutaneous *Fusarium solani* infections following VDCLP chemotherapy, highlighting the challenges in diagnosis and management.

**Case presentation:**

A 27-year-old male ALL patient developed cutaneous infections on the right calf, left upper arm, and buttock during chemotherapy. Initial lesions presented as a 1.0 × 1.0 cm dark purple nodule on the right calf, progressing rapidly to black eschar, ulceration, and multiple metastatic lesions. *Fusarium solani* was confirmed via microbiological culture and molecular testing. Treatment included systemic voriconazole, local debridement, topical liposomal amphotericin B, G-CSF for neutrophil recovery, and psychological intervention for anxiety. The infections were effectively controlled with gradual wound healing, and no recurrence was observed during 2 months of follow-up.

**Conclusion:**

Unusual skin lesions in immunocompromised patients, especially those with hematological malignancies receiving chemotherapy, warrant high suspicion for *Fusarium* infection. Timely microbiological diagnosis and early initiation of combined systemic-topical antifungal therapy, alongside neutrophil support and multidisciplinary care, are critical for improving outcomes.

## Introduction

*Fusarium* species are rare but aggressive pathogens in immunocompromised hosts, accounting for <1% of invasive fungal infections in hematological malignancies yet associated with a 40%–60% mortality rate (1, 2). In acute lymphoblastic leukemia (ALL) patients, chemotherapy-induced neutropenia significantly increases the risk of fungal infections, with *Fusarium* representing a rare but life-threatening complication (incidence: 0.5%–1.0%) (3, 4). Unlike common molds (e.g., *Aspergillus*), *Fusarium* skin infections exhibit insidious onset with atypical lesions (e.g., deep purple nodules) that rapidly progress to necrosis and ulceration, often leading to disseminated disease if untreated (5).

This case report describes a 27-year-old male ALL patient who developed multifocal cutaneous *Fusarium solani* infections during VDCLP chemotherapy, and highlights the atypical clinical presentation and diagnostic challenges, the efficacy of combined systemic voriconazole and localized amphotericin B liposome therapy, as well as the critical role of wound debridement and psychological support in managing advanced fungal skin lesions.

## Case presentation

The patient was a 27-year-old male construction worker with a junior high school education. Body weight is 65 kg, and body mass index (BMI) is 20.3 kg/m^2^, with no prior medical conditions (no history of diabetes, tumors, or prior skin infections) and no family history of genetic disorders. On admission, physical examination showed no cutaneous or mucosal lesions, but palpable mandibular lymphadenopathy with tenderness. Blood work revealed leukocytosis (124.1 × 10^9^/L, 90% blasts), anemia (Hb 78 g/L), thrombocytosis (144 × 10^9^/L), and elevated ferritin (382.3 ng/ml). Bone marrow biopsy and flow cytometry confirmed the diagnosis of acute lymphoblastic leukemia (ALL), prompting initiation of VDCLP chemotherapy (Vinorelbine, Idarubicin, Cyclophosphamide, Pegaspargase, Dexamethasone).

### Clinical course

Day 2 post-chemotherapy: Fever (39.8°C) developed alongside a 1.0 × 1.0 cm dark purplish nodule on the right calf, with mild tenderness and intact epithelium. Laboratory results showed severe neutropenia (WBC 0.5 × 10^9^/L, neutrophils 0.01 × 10^9^/L), negative fungal G/GM tests, *Klebsiella pneumoniae* bacteremia, and elevated proinflammatory cytokines (IL-6: 1165.72 pg/ml, IL-10: 215.72 pg/ml). Treatment included micafungin, meropenem, teicoplanin, and G-CSF for neutrophil recovery.

**FIGURE 1:**
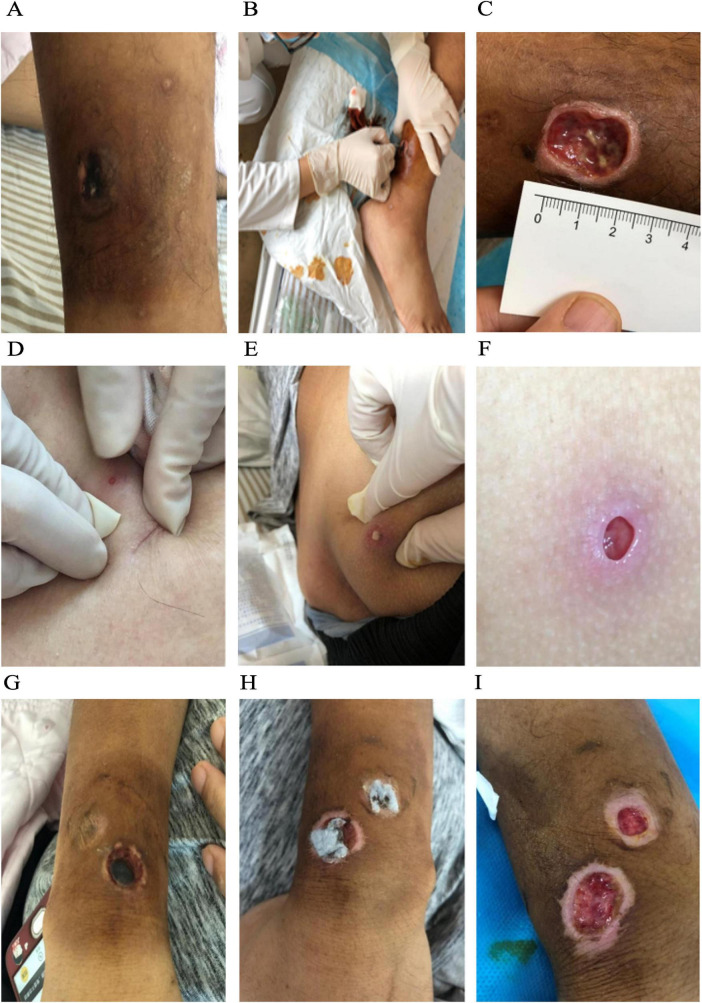
Cutaneous *Fusarium solani* infection after chemotherapy for acute lymphoblastic leukemia. Right lower leg skin infection **(A–C)**. **(A)** On the 4th day post-chemotherapy, central necrosis of the induration on the lateral aspect of the right lower leg was observed, accompanied by a black eschar; **(B)** debridement was performed for the right calf infection; **(C)** by the 17th day post-chemotherapy, the infection showed signs of resolution. Skin infection in the right buttock **(D–F)**. **(D)** on the 8th day post-chemotherapy, a 0.3 × 0.3 cm purplish nodule appeared on the right buttock; **(E)** by the 10th day post-chemotherapy, the hip induration ulcerated and formed a 0.3 × 0.3 × 1.5 cm infectious sinus tract; **(F)** by the 17th day post-chemotherapy, the infection showed signs of resolution. Skin infections in the left upper limb **(G–I)**. **(G)** On the 8th day post-chemotherapy, two pale purple hard nodules measuring 1.5 × 1.0 cm and 1.0 × 1.0 cm appeared on the left upper limb; **(H)** by the 10th day post-chemotherapy, the left upper limb’s violaceous nodule showed progressive necrosis with a central black eschar formation; **(I)** by the 17th day post-chemotherapy, the infection showed signs of resolution.

Day 4: The right calf lesion expanded to 3.0 × 3.0 cm with central necrosis and black eschar formation, accompanied by ulceration and purulent discharge at the margin (Figures 1A, B). Wound secretions were collected for culture.

Day 8: Neutrophil count improved to 3.2 × 10^9^/L, but low-grade fever persisted (37.4–37.6°C). The right calf eschar enlarged to 3.0 × 3.0 cm, and new lesions emerged: a 0.3 × 0.3 cm purplish nodule on the right buttock and two lilac nodules (1.5 × 1.0 cm and 1.0 × 1.0 cm) on the left upper limb (Figures 1D, G).

### Microbiological diagnosis

Wound culture identified *Fusarium solani*. Antifungal susceptibility showed voriconazole MIC 0.5 μg/mL (CLSI ≤ 2 μg/mL), prompting systemic therapy. Treatment was switched to oral voriconazole 200 mg bid with urgent surgical debridement of right lower extremity necrotic tissue (Figure 1B). The debridement protocol included: Local anesthesia with 1% lidocaine (reducing NRS pain score from 8 to 3); Mechanical debridement of necrotic tissue with sterile scalpel and 10% urea ointment; Irrigation with 100 ml normal saline + 10 mg amphotericin B liposome; Dressing with nano-anionic antibacterial foam (changed every 48 h).

Day 10: The right buttock nodule ulcerated into a 0.3 × 0.3 × 1.5 cm sinus tract (Figure 1E), while the left upper limb lesions developed central eschar (Figure 1H). The patient developed treatment refusal due to anxiety (HADS score: 14, DT score: 7), requiring psychological intervention with cognitive-behavioral counseling. Post-intervention scores improved to HADS 6 and DT 3, restoring treatment compliance.

### Resolution

Day 13: Afebrile.

Day 17: Neutrophils normalized (2.7 × 10^9^/L, neutrophils 1.0 × 10^9^/L). All lesions showed clinical improvement: reduced inflammation in the right calf (Figure 1C), healing of the right buttock sinus (Figure 1F), and crusting of left upper limb lesions (Figure 1I).

**FIGURE 2:**
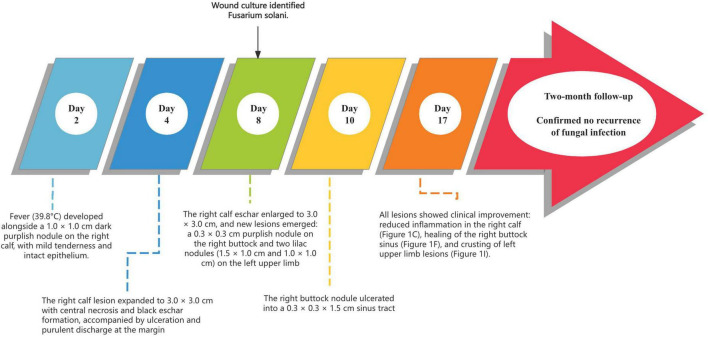
Timeline of cutaneous infection following chemotherapy in a patient with acute lymphoblastic leukemia.

The patient completed a second chemotherapy cycle uneventfully and was discharged. Two-month follow-up confirmed no recurrence of fungal infection. The complete timeline of cutaneous infection in an ALL patient from onset to recovery is shown in Figure 2.

## Discussion

*Fusarium* infections are clinically rare, with controversial routes of infection, though cutaneous involvement represents the primary site of infection and diagnostic source (3, 6). Retrospective data indicate that hematological malignancy patients carry a high risk of *Fusarium* infection (7). In immunocompromised hosts, cutaneous *Fusarium* infections exhibit insidious early onset, rapid lesion progression, and a propensity to evolve into refractory, life-threatening disseminated disease with substantial mortality (4, 8).

In immunocompromised patients, particularly those with hematological malignancies, prompt antifungal therapy is vital for managing *Fusarium* infections. Current guidelines recommend voriconazole, either alone or in combination with liposomal amphotericin B, as first-line treatment due to its proven efficacy against *Fusarium* species (9, 10). Restoring neutrophil counts is crucial, as studies have shown a direct link between neutrophil recovery and improved infection control (11, 12). Surgical debridement of infected tissues, especially cutaneous lesions, has become a key adjunctive measure, significantly reducing fungal burden and enhancing treatment outcomes (13, 14). Moreover, the patient’s psychological state plays an important role in treatment success, influencing adherence and prognosis (8).

In this case, the patient with acute lymphoblastic leukemia (ALL) exhibited profound neutropenia and immunocompromise, known risk factors for *Fusarium* infection, which were further exacerbated by chemotherapy. The treatment approach integrated systemic voriconazole and localized liposomal amphotericin B to target fungal proliferation, alongside granulocyte-colony stimulating factor (G-CSF) to accelerate neutrophil recovery. Regular skin wound debridement and antimicrobial dressing changes were performed to minimize bacterial superinfection. Notably, these interventions not only stabilized the patient’s clinical condition but also improved their psychological resilience, fostering confidence in the treatment trajectory.

While voriconazole remains the primary therapy for *Fusarium* infections, clinicians should consider alternative treatments and drug limitations. Salvage options include posaconazole for voriconazole-resistant strains or inadequate responses, supported by a study showing improved survival in refractory cases (15). Combination therapy with liposomal amphotericin B, as used in our case, exhibits *in vitro* synergistic effects and reduces fungal burden in severe infections. Limitations of voriconazole include emerging CYP51 mutations (e.g., TR34/L98H), linked to treatment failure in prolonged use, and compromised bioavailability in gastrointestinal dysfunction (e.g., chemotherapy-induced mucositis), necessitating therapeutic drug monitoring. Tailored strategies and close clinical follow-up are critical for optimizing outcomes in high-risk patients (9).

*Fusarium* infections are characterized by rapid progression, frequently manifesting as suppurative lesions and necrotic eschar. This necessitates immediate and thorough physical examinations to identify infected sites promptly (8, 16). In our case, the initial manifestation of skin involvement was a purplish nodule, which, coupled with negative results from blood cultures, fungal (1-3)-β-D glucan (G) test, and *Aspergillus* galactomannan (GM) test, posed significant challenges for early diagnosis. It was only through microbiological analysis of wound secretions obtained from the ulcerated skin that a definitive diagnosis of *Fusarium* was established (14). This highlights the critical role of timely and accurate microbiological testing in guiding appropriate treatment strategies for *Fusarium* infections (13, 17).

## Conclusion

In conclusion, prompt microbiological testing is essential for managing *Fusarium* skin infections, especially in hematological chemotherapy patients. Early diagnosis allows for timely systemic and local antifungal treatment, combined with neutrophil–boosting measures and debridement. A multidisciplinary approach, including psychological support, improves patient compliance and outcomes, highlighting the need for comprehensive care in high–risk groups.

## Data Availability

To protect patient privacy, patient-related data such as pathology image datasets and other patient-related data are not publicly accessible, but all data can be obtained upon reasonable request to QA, aiqinqin.zju@foxmail.com. To gain access, data requesters will need to sign a data-access agreement.
